# Synthesis and Evaluation of ^18^F-labeled Pyridaben Analogues for Myocardial Perfusion Imaging in Mice, Rats and Chinese mini-swine

**DOI:** 10.1038/srep33450

**Published:** 2016-09-20

**Authors:** Tiantian Mou, Zuoquan Zhao, Linyi You, Yesen Li, Qian Wang, Wei Fang, Jie Lu, Cheng Peng, Xianzhong Zhang

**Affiliations:** 1 Center for Molecular Imaging and Translational Medicine, State Key Laboratory of Molecular Vaccinology and Molecular Diagnostics, School of Public Health, Xiamen University, Xiamen 361005, China; 2 Department of Nuclear Medicine, Beijing Anzhen Hospital, Capital Medical University, Beijing 100029, China; 3 Department of Nuclear Medicine, Cardiovascular Institute and Fu Wai Hospital, Chinese Academy of Medical Sciences, Beijing 100037, China; 4 Key Laboratory of Radiopharmaceuticals, Ministry of Education, College of Chemistry, Beijing Normal University, Beijing 100875, China; 5 PET Center, Xuanwu Hospital of Capital University of Medical Sciences, Beijing, 100053, China

## Abstract

This study reports three novel ^18^F-labeled pyridaben analogues for potential myocardial perfusion imaging (MPI). Three precursors and the corresponding nonradioactive compounds were synthesized and characterized. The radiolabeled tracers were obtained by substituting tosyl with ^18^F. The total radiosynthesis time of these tracers was 70–90 min. Typical decay-corrected radiochemical yields were 47–58%, with high radiochemical purities (>98%). Tracers were evaluated as MPI agents *in vitro*, *ex vivo* and *in vivo*. In the mouse biodistribution study, all three radiotracers showed high initial heart uptake (34–54% ID/g at 2 min after injection) and fast liver clearance. In the microPET imaging study, [^18^F]Fmpp2 produced heart images with good quality in both mice and rats. In the whole-body PET/CT images of mini-swine, [^18^F]Fmpp2 showed excellent initial heart standardized uptake value (SUV) (7.12 at 5 min p.i.) and good retention (5.75 at 120 min p.i.). The heart/liver SUV ratios were 4.12, 5.42 and 5.99 at 30, 60 and 120 min after injection, respectively. The favorable biological properties of [^18^F]Fmpp2 suggest that it is worth further investigation as a potential MPI agent.

Myocardial perfusion imaging (MPI) is a non-invasive method in the diagnostic and prognostic evaluation of coronary artery disease (CAD)^1^. Although ^201^Tl and ^99 m^Tc-sestamibi are the mainstay of MPI, they still have some defects^2^. Positron emission tomography (PET) technology offers a better resolution and effective correction of photo attenuation and scatter, leading to absolute quantification of regional myocardial blood flow and coronary flow reserve^3^,^4^. Thus, researchers are devoted to the development of clinical PET MPI agents, such as ^13^N-NH_3,_^15^O-H_2_O, and ^82^Rb^5^,^6^. ^18^F has a much longer half-life than ^13^N, ^15^O and ^82^Rb, making it more convenient for clinical use. In the last decade, several ^18^F-labeled lipophilic cations (^18^F-FBnTP^7^,^8^, ^18^F-FERhB^9^, ^18^F-FTPP^10^,^11^, ^18^F-FPTP^12^, ^18^F-3^13^, [^18^F]FMBTP^14^) and analogues of mitochondrial complex I (MC-I) inhibitors (^18^F-FDHR^15^, ^18^F-RP1004^16^, BMS-747158-02^17^,^18^,^19^) have been reported. MC-I, which located in the inner mitochondrial membrane^20^, is an ideal MPI target. The heart uptake of MC-I inhibitors, such as rotenone, quinazoline, and pyridaben, is correlated with the myocardial blood flow. BMS-747158-02 (a pyridaben analogue, commercially branded as Flurpiridaz F 18) has been applied in phase III clinical studies^21^.

Previously, our group reported a series of pyridaben analogues as potential MPI agents^22^,^23^,^24^,^25^. [^18^F]FP1OP exhibits the most promising biological properties with high initial heart uptake and good target-to-background ratios. However, its moderate instability in water limits its possibility to be an ideal MPI agent^23^. The following compounds [^18^F]FPTP2^22^ and [^18^F]Fmp2^25^ have excellent stabilities. However, their liver clearance is slow, and thus the heart/liver ratios are not ideal. On the basis of our experience, PEGylated pyridaben analogues have fast liver clearance^22^,^23^. Thus, in this study, we modified the structure of [^18^F]Fmp2 with polyethylene glycol (PEG) chains. Herein, we report the synthesis of three novel ^18^F-labeled pyridaben analogues, 4-chloro-2-tert-butyl-5-[2-[[1-[2-[^18^F]fluroethoxymethyl]-1H-1,2,3-triazol-4-yl]methyl]phenylmethoxy]-3(2H)-pyridazinone ([^18^F]Fmpp1), 4-chloro-2-tert-butyl-5-[2-[[1-[2-[2-[^18^F]fluroethoxy]ethoxymethyl]-1H-1,2,3-triazol-4-yl]methyl]phenylmethoxy]-3(2H)-pyridazinone ([^18^F]Fmpp2), and 4-chloro-2-tert-butyl-5-[2-[[1-[2-[2-[2-[^18^F]fluroethoxy]ethoxy]ethoxymethyl]-1H-1,2,3-triazol-4-yl]methyl]phenylmethoxy]-3(2H)-pyridazinone ([^18^F]Fmpp3), and the evaluation of their *in vitro* physicochemical properties, *ex vivo* biodistribution in mice and *in vivo* PET imaging properties in mice, rats and Chinese mini-swine.

## Results

### Chemistry

**Figure 1:**
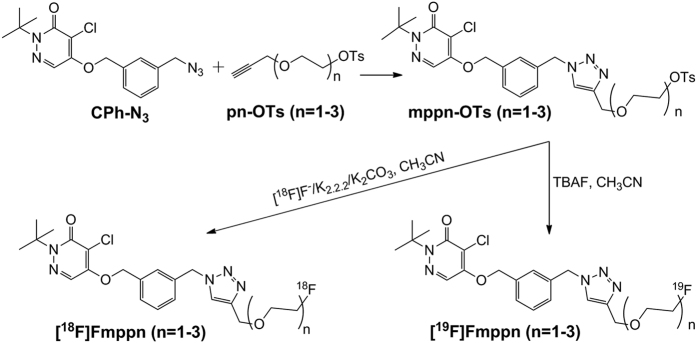
The synthesis route of [^18/19^F]Fmppn (n = 1–3).

The precursors 4-chloro-2-tert-butyl-5-[3-[[1-[2-[(4-methylphenyl)sulfonyloxy]ethoxymethyl]-1H-1,2,3-triazol-4-yl]methyl]phenylmethoxy]-3(2H)-pyridazinone (mpp1-OTs), 4-chloro-2-tert-butyl-5-[3-[[1-[2-[2-[(4-methylphenyl)sulfonyloxy]ethoxy]ethoxymethyl]-1H-1,2,3-triazol-4-yl]methyl]phenylmethoxy]-3(2H)-pyridazinone (mpp2-OTs), 4-chloro-2-tert-butyl-5-[3-[[1-[2-[2-[2-[(4-methylphenyl)sulfonyloxy]ethoxy] ethoxy]ethoxymethyl]-1H-1,2,3-triazol-4-yl]methyl]phenylmethoxy]-3(2H)-pyridazinone (mpp3-OTs) and corresponding non-radioactive references [^19^F]Fmpp1, [^19^F]Fmpp2 and [^19^F]Fmpp3 were synthesized according to the general procedure (Fig. 1). The yields of the precursors mpp1-OTs, mpp2-OTs, mpp3-OTs were 64%, 68% and 58%, respectively. The yields of non-radioactive references [^19^F]Fmpp1, [^19^F]Fmpp2 and [^19^F]Fmpp3 were 42%, 47% and 44%, respectively. Compounds were characterized by ^1^H NMR, ^13^C NMR, ^19^F NMR, ESI-MS and IR (the data were shown in the Supplementary File). The chemical purities of [^19^F]Fmpp1, [^19^F]Fmpp2 and [^19^F]Fmpp3 were calculated as more than 95% from the high performance liquid chromatography (HPLC) analysis (λ = 254 nm), suggesting they could be acceptable referenced standards for the corresponding radioactive tracers.

### Radiochemistry

**Figure 2:**
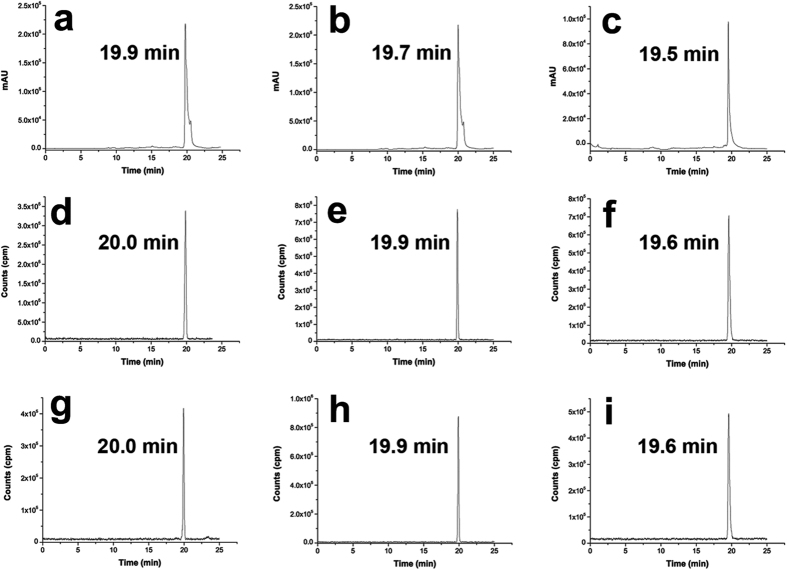
HPLC chromatograms of compounds [^19^F]Fmpp1 (**a**), [^19^F]Fmpp2 (**b**) [^19^F]Fmpp3 (**c**), [^18^F]Fmpp1 (**d**), [^18^F]Fmpp2 (**e**), [^18^F]Fmpp3 (**f**), and profiles of [^18^F]Fmpp1 (**g**), [^18^F]Fmpp2 (**h**) [^18^F]Fmpp3 (**i**) after storage in water at room temperature for 3 h. Non-radioactive compounds of [^19^F]Fmpp1 (**a**), [^19^F]Fmpp2 (**b**) [^19^F]Fmpp3 (**c**) were measured with a UV detector, and radioactive compounds were measured radiometrically.

Starting from the [^18^F]KF/K_222_ solution, the total synthesis time was 70–90  min, including HPLC purification. The radiochemical yields (RCYs) of [^18^F]Fmpp1, [^18^F]Fmpp2 and [^18^F]Fmpp3 were 55 ± 9.2%, 58 ± 7.1%, and 47 ± 6.4% with decay correction. Their identification was confirmed by co-injecting with corresponding non-radioactive references onto a HPLC. The retention time of [^18^F]Fmpp1, [^18^F]Fmpp2 and [^18^F]Fmpp3 were 20.0 min, 19.9 min and 19.6 min, respectively, consistent with the corresponding references (Fig. 2). The RCPs of three tracers calculated from radio-HPLC chromatogram were over 98% after purification. The specific activities were 20–40 GBq/μmol at the end of synthesis as determined by HPLC analysis.

### Physicochemical properties study

The partition coefficient (log P) values of [^18^F]Fmpp1, [^18^F]Fmpp2 and [^18^F]Fmpp3 were 1.98 ± 0.03, 1.73 ± 0.05 and 1.54 ± 0.14, respectively (n = 3). The log P values were similar to [^18^F]FP2OP and [^18^F]FP3OP^22^,^23^, indicating that they were lipophilic compounds. Stability studies in water revealed that all three tracers showed no significant change in radiochemical purities, suggesting that they were stable in water for at least 3 h. When incubated in murine plasma at 37 °C for 2 h, approximately 40% [^18^F]Fmpp1 was decomposed, while the other two tracers remained intact.

### Biodistribution in mice

As shown in Table 1, [^18^F]Fmpp1, [^18^F]Fmpp2 and [^18^F]Fmpp3 had high initial heart uptake (54.82 ± 5.74, 42.38 ± 4.40 and 34.01 ± 3.54% ID/g at 2 min post-injection (p.i.), respectively) and good target/non-target ratios. All three tracers showed fast liver clearance (approximately 36%, 47% and 54% of the radioactivity was cleared from liver for [^18^F]Fmpp1, [^18^F]Fmpp2 and [^18^F]Fmpp3, respectively, at 60 min p.i. compared with that at 2 min p.i.). However, all three tracers were washed out quickly from the heart (approximately 82%, 57% and 62% of the radioactivity was cleared from the heart for [^18^F]Fmpp1, [^18^F]Fmpp2 and [^18^F]Fmpp3, respectively, at 60 min p.i. compared with that at 2 min p.i.), which affected the heart/liver ratios at subsequent time points. Among the three radiotracers, [^18^F]Fmpp2 showed the best biological properties. Its heart/liver, heart/lung and heart/blood ratios were higher than 3.5 at all time points, encouraging further evaluation.

All three radiotracers exhibited distinct uptake in muscle, which might be due to the high mitochondrial expression in calf muscle. Low bone uptake was observed in [^18^F]Fmpp2 and [^18^F]Fmpp3, indicating no defluorination of those tracers *in vivo.* The relative increase in [^18^F]Fmpp1 in bone uptake might be due to its instability *in vivo*, which is consistent with the stability study. All three radiotracers had notable uptake in the kidneys, indicating that these tracers were excreted via the renal system.

### Whole-body PET/CT imaging in Chinese mini-swine

**Figure 3:**
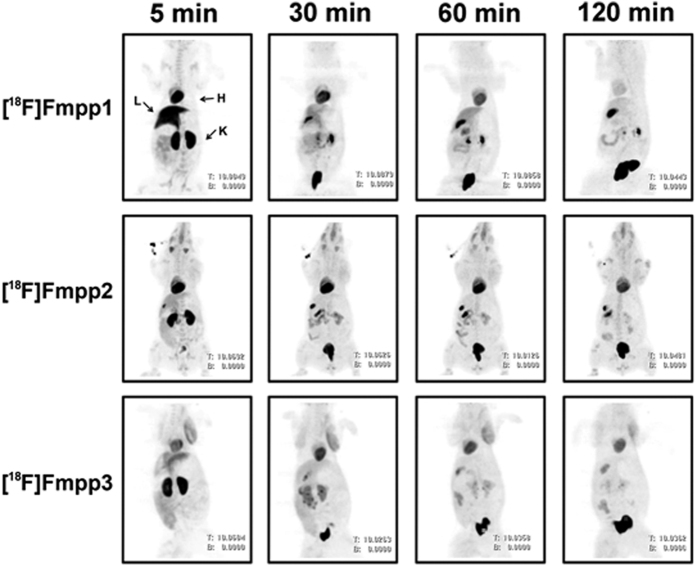
Whole-body MIP PET/CT images of healthy Chinese mini-swine. Images were obtained at 5, 30, 60, and 120  min after injection approximately 37 MBq of [^18^F]Fmpp1, [^18^F]Fmpp2 and [^18^F]Fmpp3 in 5% ethanol solution. H = heart; L = liver; K = kidney.

The behavior of tracers in Chinese mini-swine is different from that in mice. All three tracers showed high initial heart uptake in Chinese mini-swine (Fig. 3). The standardized uptake values (SUVs) of [^18^F]Fmpp1, [^18^F]Fmpp2 and [^18^F]Fmpp3 were 6.82, 7.12 and 4.51 at 5 min p.i. (Table S1 of Supplementary file). At 60 min p.i., the heart uptake of [^18^F]Fmpp2 and [^18^F]Fmpp3 decreased 19% and 0% compared with the data at 5 min p.i., indicating remarkable retention in the heart. In addition, all three tracers were quickly washed out from the liver. At 30 min p.i., the liver uptake of [^18^F]Fmpp2 and [^18^F]Fmpp3 was 59% and 48% lower than that at 5 min p.i. Furthermore, there was low uptake in other non-target organs and tissues, except the gallbladder, kidneys and bladder.

Among these three tracers, [^18^F]Fmpp2 provides images with the best quality. Its heart/liver SUV ratios were 1.72, 4.12, 5.42 and 5.99 at 5, 30, 60 and 120 min, respectively (Table S1 of Supplementary file). In addition, the low uptakes and fast clearance were observed for non-target organs. The SUVs of [^18^F]Fmpp2 in most of the non-target tissues were negligible after 30 min p.i., including the liver, lung, kidneys and blood. As shown in Fig. 3, the heart can be observed clearly from 5 min to 120 min p.i., almost without any interruption from the nearby tissues. Thus, we could obtain quality images within a wide range of time (from 30–120 min p.i.), which is convenient for diversified clinical imaging protocols.

The images of [^18^F]Fmpp3 were also commendable. Its early heart uptake was not as outstanding as [^18^F]Fmpp2. However, it had preferable retention in the heart (from 4.51 at 5 min p.i. to 4.53 at 120 min p.i., SUVs) and faster liver clearance (from 4.10 at 5 min p.i. to 1.12 at 120 min p.i., SUVs). The heart/liver SUV ratios were 2.56, 3.98 and 4.04 at 30, 60, and 120 min p.i., respectively (Table S1 of Supplementary file). Accordingly, the images obtained during 30–120 min p.i. had good signal-noise ratios. However, although the other tracer [^18^F]Fmpp1 also had high initial heart SUV (6.82 at 5 min p.i.), the clearance from the heart (2.19 at 120 min p.i., SUV) was much faster than the other two tracers. Thus, the outline of the heart was not as clear as the other tracers of [^18^F]Fmpp2 and [^18^F]Fmpp3 at subsequent time points.

All three tracers had notable initial uptake in the kidneys. The radioactivities were quickly washed out to the bladder, indicating that they were excreted mainly via the renal system. This result was consistent with that of the mouse biodistribution. No obvious bone uptake was observed in all three tracers, indicating that there was no defluorination in Chinese mini-swine. Taken together, these findings suggested that [^18^F]Fmpp2 had the potential for MPI and is worth further evaluation.

### MicroPET/CT studies in mice and rats

**Figure 4:**
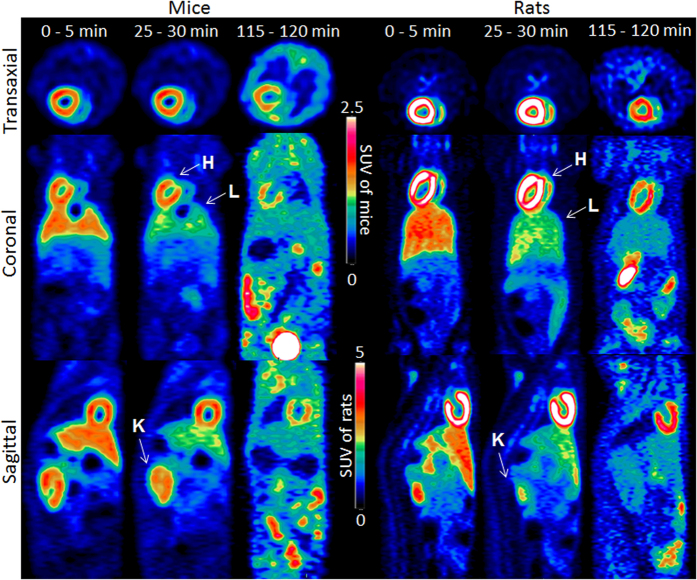
MicroPET images in wildtype mice and rats. Images were obtained at 5, 30 and 120 min after injection of [^18^F]Fmpp2 in 5% ethanol solution (10 MBq per mouse and 35 MBq per rat). H = heart; L = liver; K = kidney.

To visualize the distribution of [^18^F]Fmpp2 in different animal species, microPET imaging studies were performed in mice and rats (Fig. 4). [^18^F]Fmpp2 had notable heart uptake in the mice at 5 and 30 min p.i. However, most of the radioactivities were washed out from the heart at 120 min p.i., which was coincident with the result of the biodistribution study. The liver clearance of [^18^F]Fmpp2 was faster than that of the heart (Table S2 of Supplementary file). Thus, we could obtain clear images at 30 min p.i., with a good heart/liver ratio (1.52). The lower heart/liver ratios in microPET images compared to the biodistribution study may be due to different injection doses and quantification methods.

From the microPET imaging results of rats (Fig. 4), [^18^F]Fmpp2 had much higher heart uptake and better retention than that of mice (Table S2 of Supplementary file). At 120 min p.i., only approximately 43% radioactivity was cleared from the heart compared with that at 5 min p.i. The heart clearance of rats was much slower than that of mice (74% at the same condition), resulting in higher heart/blood, heart/liver, heart/lung and heart/muscle ratios. The outline of the heart was still visible at 120 min p.i.

In general, microPET imaging of [^18^F]Fmpp2 in both mice and rats could provide legible images at 30 min p.i. There was specific uptake in the kidneys of both mice and rats, indicating that the kidneys were the main organs for excretion.

### Metabolic stability

**Figure 5:**
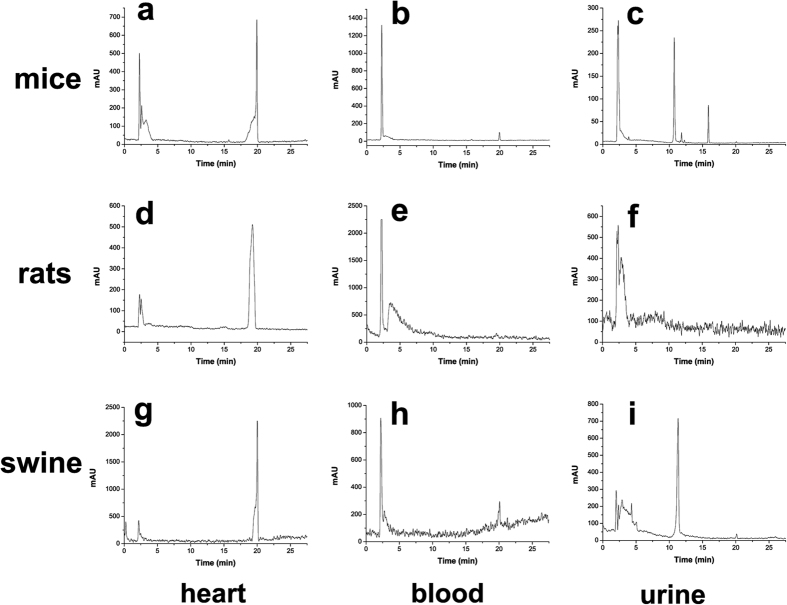
The HPLC profiles of metabolites of [^18^F]Fmpp2 in wildtype Kunming mice, rats and Chinese mini-swine. Soluble fractions were collected in heart (**a,d,g**), blood (**b,e,h**) and urine (**c,f,i**) at 60 min p.i.

The metabolic stabilities of [^18^F]Fmpp2 were investigated in mice, rats and Chinese mini-swine. As shown in Fig. 5, [^18^F]Fmpp2 had much better stability in the hearts of rat and Chinese mini-swine than that of mice. According to the HPLC analysis of the extracts from hearts of mouse, rat and Chinese mini-swine, approximately 45%, 13% and 13% activities were metabolized into more hydrophilic compounds at 60 min p.i., respectively. In blood and urine samples, the peak of [^18^F]Fmpp2 almost disappeared at 60 min p.i. in all animal models using HPLC analysis, suggesting that the tracer was transformed into more hydrophilic compounds in both the blood and urine of mouse, rat and Chinese mini-swine.

## Discussion

Compared with previously reported compounds of [^18^F]FP1OP^23^ and [^18^F]FP2OP^24^, [^18^F]Fmpp2 had better stability in a water solution, which is propitious to the long-distance transport. In addition, [^18^F]Fmpp2 has higher initial heart uptake and better retention in Chinese mini-swine. Thus, the signal-noise ratios of [^18^F]Fmpp2 are more ideal, particularly at subsequent time points. Compared with [^18^F]Fmp2^25^, the PEGylated tracer [^18^F]Fmpp2 has obvious faster liver clearance, which is consistent with our previous reports^22^,^23^. For [^18^F]Fmp2, its liver uptake in mice increased with time (55% increase at 60 min p.i. from 2 min p.i., while [^18^F]Fmpp2 demonstrated a 49% decrease in mouse liver uptake at 60 min p.i. from 2 min p.i. under the same conditions. These results indicate that the PEG chain is an effective functional group for the design of pyridaben analogues for MPI.

In this study, [^18^F]Fmpp2 showed the most promising properties as a potential MPI agent among the three radiotracers. When compared with the well-known MPI agent Flurpiridaz F 18^26^, [^18^F]Fmpp2 has much faster liver clearance, and thus it can provide better quality images earlier (15–30 min p.i.) compared to flurpiridaz (after 60 min p.i.). The wider range of imaging time (15–120 min p.i.) is more convenient for diversified clinical imaging protocols. In addition, as shown in Fig. 3, the radioactivities were washed out extremely quickly from non-target tissues, including the kidneys, which can definitely lower the irradiation dose.

The different behaviors of [^18^F]Fmpp2 in mice, rats and Chinese mini-swine may be due to its metabolic stability in different animal species. In the heart of mice, 45% activities of [^18^F]Fmpp2 were metabolized at 60 min p.i., accompanied by 57% activities of [^18^F]Fmpp2 washed out from the heart at the same time. Fortunately, the heart clearance of [^18^F]Fmpp2 was much slower in rats and Chinese mini-swine. This may due to the better stabilities in the hearts of rat and Chinese mini-swine. Only 13% activities of [^18^F]Fmpp2 were metabolized in both the hearts of rat and Chinese mini-swine at 60 min p.i., and 43% and 19% activities washed out from the hearts of rat and Chinese mini-swine, respectively, at 120 min p.i. (compared to the data at 5 min p.i.). Further studies, such as imaging with ischemia animal models and evaluation with microspheres, are needed in the future.

## Methods

[^18^F]fluoride trapped on a Sep-pak QMA cartridge (Waters Inc. USA) was obtained from the PET Center of Xuanwu Hospital (Beijing, China). 4-chloro-2-tert-butyl-5-[[3-(azidomethyl)phenyl]methoxy]-3(2H)-pyridazinone (CPh-N_3_), 2-(2-propyn-1-yloxy)ethoxy-1-(4-methylbenzenesulfonate) ethanol (P1-OTs), 2-(2-(2-propyn-1-yloxy)ethoxy)-1-(4-methylbenzenesulfonate) ethanol (P2-OTs) and 2-(2-(2-(2-propyn-1-yloxy)ethoxy)ethoxy)-1-(4-methylbenzenesulfonate) ethanol (P3-OTs) was synthesized in the Key Laboratory of Radiopharmaceuticals of Beijing Normal University according to a published procedure^22^. Other reagents and solvents were purchased from commercial suppliers. The reversed-phase HPLC analysis for metabolic stability studies was performed on a Waters 2535Q system with a Gabi-star flow-counter (Raytest Inc. Germany). HPLC for other studies were performed on an SHIMADZU system with LC-20AT pumps and a B-FC-320 BIOSCAN flow-counter. A C-18 reverse-phase semi-preparative HPLC column (10 × 250 mm, 5-μm particle size, Venusil MP-C18) was purchased from Agela Technologies Inc. (USA). A Labgen 7 homogenizer was purchased from Cole-Parmer Instrument (USA). ^1^H NMR spectra were recorded on a Bruker (400 MHz) spectrometer, while ^13^C NMR spectra were recorded on a Bruker (100 MHz) spectrometer. Chemical shifts are reported in δ (ppm) values. Infrared spectra were measured on a Nicolet 360 Avatar instrument, scanning from 400 to 4000 cm^−1^. Mass spectra were recorded using a Brucker Apex IV FTM instrument.

Kunming mice (18–20 g) and rats (approximately 200 g) were obtained from the Animal Center of Peking University for biodistribution and metabolic stability studies. Kunming mice (approximately 23 g) and rats (approximately 220 g) were obtained from Xiamen University Laboratory Animal Center for microPET imaging. Healthy Chinese mini-swine (approximately 15 kg) were obtained from the Animal Center of Fu Wai Hospital. All animal studies were performed under a protocol approved by the Beijing Administration Office of Laboratory Animal (BAOLA).

### Chemistry

Three tosylated precursors mpp1-OTs, mpp2-OTs and mpp3-OTs were synthesized according to the reported procedure^27^. Water (4 mL) was added to the mixture of CuSO_4_·5H_2_O (0.37 g, 1.5 mmol) and L-sodium ascorbate (1.19 g, 6 mmol) under protection of nitrogen. After stirring for 30 min, CPh-N_3_ (1.03 g, 3 mmol in 1.5 mL DMF) and P1-OTs (or P2-OTs, P3-OTs) (3 mmol in 3 mL DMF) was added and stirred for an additional 24 h. Next, the reaction solution was diluted with CH_2_Cl_2_ (100 mL), and washed with saturated NaCl solution (100 mL). The organic phase was separated and dried with Na_2_SO_4_, filtered, and then concentrated under reduced pressure. The residue was chromatographed over a column of silica gel and eluted with a mixture of hexane and ethyl acetate = 1:2 (v/v) (1:3 for mpp2-OTs, 1:4 for mpp3-OTs). The precursors of mpp1-OTs (or mpp2-OTs, mpp3-OTs) were obtained as a white solid.

The solution of tert-butylammonium fluoride (1 mmol in 1 mL tetrahydrofuran) was stirred in a stream of nitrogen at 110 °C to remove the solvent. Next, mpp1-OTs (or mpp2-OTs, mpp3-OTs) (0.30 mmol in 3 mL anhydrous CH_3_CN) were added and refluxed for 2 h. After concentration under reduced pressure, the residue was chromatographically separated over a column of silica gel and eluted with a mixture of hexane and ethyl acetate = 1:2 (v/v) for [^19^F]Fmpp1 (1:3 for [^19^F]Fmpp2 and 1:4 for [^19^F]Fmpp3). Final compounds of [^19^F]Fmpp1 (or [^19^F]Fmpp2 and [^19^F]Fmpp3) were obtained as a brown oil.

### Radiochemistry

The radiolabeling procedure of [^18^F]Fmpp1, [^18^F]Fmpp2 and [^18^F]Fmpp3 was performed as previously described (Fig. 1)^22^. Briefly, the solvent of [^18^F]fluoride eluant was removed at 110 °C. Tosylate precursor (2 mg in 1 mL anhydrous acetonitrile) was added and stirred for 20 min at 90 °C. The reaction solution was injected onto a semi-HPLC column, and eluted with water (solvent A) and acetonitrile (solvent B) at a flow rate of 5.0 mL/min. Gradient: 0–5 min: 95% A, 5.01–8 min: 95-60% A, 8.01–19 min: 60–30% A, 19.01–30 min: 0% A. The desired product was collected and the solvent was evaporated using a rotary evaporator. The product was re-dissolved in 5% ethanol solution, and filtered through a 0.22-μm Millipore filter.

The final RCPs were determined by re-injection of the product onto a radio-HPLC. The radioactive fraction was collected and measured in a dose calibrator for specific activity calculation. The mass of the product was calculated by comparing the area under the UV curve at 254 nm with that of a standard reference.

### Physicochemical properties studies

The octanol/water partition coefficient was measured using a shake-flask method as previously described^28^. In the study of vitro stabilities^29^, radiotracers were incubated in water at room temperature for 3 h, or in 0.5 mL murine plasma at 37 °C for 2 h, respectively. Plasma proteins were precipitated by the addition of 100 μL acetonitrile and removed by centrifugation. Next, the RCPs were assayed using radio-HPLC.

### Biodistribution study

Biodistribution studies were carried out in accordance with the approved guidelines. Approximately 185 kBq radiotracer ([^18^F]Fmpp1, [^18^F]Fmpp2 and [^18^F]Fmpp3) in 0.1 mL 5% ethanol solution was injected through the tail vein of wildtype Kunming mice (n = 5). The mice were sacrificed at 2, 15, 30 and 60 min p.i. The heart, liver and other organs of interest were collected, weighed and measured with a gamma counter. The percentage of injected dose per gram (% ID/g) for each sample was calculated, and the values were expressed as the mean ± standard deviation (SD).

### Whole-body PET/CT of Chinese mini-swine

PET/CT imaging studies of mini-swine were carried out in accordance with the approved guidelines. Healthy Chinese mini-swine were anesthetized^23^ and placed prone on the PET/CT bed. A venous catheter was established for radiotracer injection. Approximately 37 MBq of radiotracer ([^18^F]Fmpp1, [^18^F]Fmpp2 or [^18^F]Fmpp3) in 2 mL of 5% ethanol solution was injected intravenously. Whole-Body imaging was performed on a PET/CT system (Biograph 64, Siemens Healthcare) at 5, 30, 60, and 120 min p.i. Whole-Body scanning involved 4 bed positions, with each scan lasting for 2 min. Regions of interest were drawn on the myocardium and liver. SUVs were calculated as [mean region-of-interest count (cps/pixel) × body weight (kg)]/[injected dose (mCi) × calibration factor (cps/pixel)]. The myocardium/liver SUV ratios at each time point were evaluated.

### MicroPET/CT studies in mice and rats

PET/CT imaging studies of mice and rats were carried out in accordance with the approved guidelines. For microPET imaging studies, mice were injected with approximately 10 MBq of [^18^F]Fmpp2 via the tail vein (n = 4 for each group). For static scans, the mice were anesthetized with isoflurane (5% for induction and 2% for maintenance in pure oxygen gas) using a knockdown box at 5, 30 and 120 min p.i. With the help of a laser beam attached to the scanner, the mice were placed in the prone position near the center of the FOV of the scanner. Static microPET images were obtained within 5 min after whole body CT scan (6 min). The images were reconstructed using ordered subset expectation maximization with three-dimensional resolution recovery (OSEM 3D) with CT-based attenuation correction, and scatter correction. For data analysis, the region of interest (ROI) was manually drawn and covered the entire target on the CT images. This ROI was copied to the corresponding PET images. The mean SUV of the heart, liver and lung in the ROIs was recorded.

For the dynamic scan, the acquisition began at 30 s after injection and continued for 30 min. The microPET and CT images were generated separately and then fused using Inveon Research Workplace (Siemens). The images were reconstructed using ordered subset expectation maximization with three-dimensional resolution recovery (OSEM 3D) with CT-based attenuation correction, and scatter correction. The reconstruction frames were 12 × 5, 8 × 30, 5 × 300 s for dynamic imaging. For data analysis, the region of interest (ROI) was manually drawn and covered the entire target on the CT images. This ROI was copied to the corresponding PET images. The mean SUVs of the heart, liver and lung in the ROIs were recorded.

MicroPET/CT imaging in rats were also performed according to the same procedure described above with more activities (approximately 35 MBq per rat).

### Metabolic stability

Metabolic studies were carried out in accordance with the approved guidelines. Wildtype Kunming mice, rats and Chinese mini-swine were intravenously injected with 3.7 MBq, 18.5 MBq and 74 MBq [^18^F]Fmpp2, respectively. Mice and rats were sacrificed at 60 min p.i. Blood, urine and heart samples were collected as previously described^30^. Chinese mini-swine was anesthetized^23^. At 60 min p.i., blood was collected from the superior vena cava. Urine was collected from the bladder. Heart sample was collected from the anterior wall of the heart. All the samples were treated as previously described^30^. HPLC analysis was performed on a Waters 2535Q system with a Gabi-star flow-counter (Raytest Inc.) to improve the signal-noise ratios. The retention time of [^18^F]Fmpp2 was confirmed by co-injecting with [^19^F]Fmpp2 onto a HPLC.

## Additional Information

**How to cite this article**: Mou, T. *et al.* Synthesis and Evaluation of ^18^F-labeled Pyridaben Analogues for Myocardial Perfusion Imaging in Mice, Rats and Chinese mini-swine. *Sci. Rep.* **6**, 33450; doi: 10.1038/srep33450 (2016).

## Supplementary Material

Supplementary Information

## Figures and Tables

**Table 1:** The biodistribution results of [^18^F]Fmpp1, [^18^F]Fmpp2, and [^18^F]Fmpp3 in wildtype mice (expressed as % ID/g ± SD, n = 5).

Tissues	Radiotracers	Post-injection time (min)			
		2	15	30	60
Heart	[^18^F]Fmpp1	54.82 ± 5.74	22.32 ± 1.26	15.68 ± 0.95	10.00 ± 1.41
	[^18^F]Fmpp2	42.38 ± 4.40	36.42 ± 4.74	27.15 ± 3.58	18.20 ± 2.31
	[^18^F]Fmpp3	34.01 ± 3.54	21.12 ± 2.09	17.19 ± 2.58	13.00 ± 1.45
Liver	[^18^F]Fmpp1	12.34 ± 2.03	12.00 ± 0.41	9.37 ± 0.21	7.91 ± 1.17
	[^18^F]Fmpp2	9.93 ± 1.08	8.34 ± 1.32	6.85 ± 1.89	5.10 ± 1.25
	[^18^F]Fmpp3	8.74 ± 1.56	4.45 ± 0.24	4.31 ± 0.87	4.02 ± 0.33
Spleen	[^18^F]Fmpp1	3.32 ± 0.69	4.45 ± 0.51	4.00 ± 0.15	3.74 ± 0.44
	[^18^F]Fmpp2	3.67 ± 0.67	4.35 ± 1.10	3.49 ± 0.74	3.67 ± 0.38
	[^18^F]Fmpp3	4.07 ± 1.63	4.19 ± 0.32	3.93 ± 0.67	3.96 ± 0.26
Lung	[^18^F]Fmpp1	6.78 ± 0.22	4.80 ± 0.29	4.82 ± 0.30	4.56 ± 0.37
	[^18^F]Fmpp2	9.46 ± 1.14	4.66 ± 2.70	3.70 ± 0.27	4.21 ± 0.46
	[^18^F]Fmpp3	8.18 ± 1.97	4.68 ± 0.49	4.42 ± 0.61	4.69 ± 0.50
Muscle	[^18^F]Fmpp1	14.99 ± 1.42	9.19 ± 1.13	8.31 ± 0.24	6.29 ± 1.27
	[^18^F]Fmpp2	10.13 ± 3.73	11.45 ± 2.71	8.41 ± 1.44	8.25 ± 1.30
	[^18^F]Fmpp3	11.96 ± 1.74	9.85 ± 0.93	9.40 ± 1.35	10.00 ± 1.74
Bone	[^18^F]Fmpp1	5.97 ± 1.73	6.59 ± 0.95	7.42 ± 0.30	10.79 ± 1.72
	[^18^F]Fmpp2	5.82 ± 0.44	5.03 ± 0.42	4.10 ± 0.56	5.07 ± 0.46
	[^18^F]Fmpp3	7.22 ± 1.95	4.54 ± 0.56	4.05 ± 1.14	6.48 ± 1.33
Kidney	[^18^F]Fmpp1	16.70 ± 5.13	17.76 ± 1.19	14.94 ± 0.98	7.19 ± 0.93
	[^18^F]Fmpp2	24.61 ± 4.16	20.32 ± 3.54	14.20 ± 1.99	10.50 ± 0.94
	[^18^F]Fmpp3	26.63 ± 5.72	12.79 ± 1.16	9.98 ± 1.50	7.42 ± 0.79
Blood	[^18^F]Fmpp1	2.93 ± 0.19	3.55 ± 0.43	4.32 ± 0.15	5.48 ± 0.56
	[^18^F]Fmpp2	1.39 ± 0.26	2.17 ± 0.32	2.64 ± 0.54	3.65 ± 0.53
	[^18^F]Fmpp3	3.31 ± 0.61	4.69 ± 0.51	4.50 ± 0.98	5.03 ± 1.39
Heart/Liver	[^18^F]Fmpp1	4.44	1.86	1.67	1.27
	[^18^F]Fmpp2	4.27	4.37	3.96	3.57
	[^18^F]Fmpp3	3.89	4.75	3.98	3.23
Heart/Lung	[^18^F]Fmpp1	8.09	4.65	3.25	2.19
	[^18^F]Fmpp2	4.48	7.82	7.34	4.32
	[^18^F]Fmpp3	4.16	4.51	3.89	2.77
Heart/Blood	[^18^F]Fmpp1	18.73	6.28	3.63	1.83
	[^18^F]Fmpp2	30.52	16.77	10.29	4.98
	[^18^F]Fmpp3	10.26	4.51	3.82	2.58
