# Supplementary material for: Synthesis and Evaluation of 18F-labeled Pyridaben Analogues for Myocardial Perfusion Imaging in Mice, Rats and Chinese mini-swine
Source: Sci Rep. 2016 Sep 20;6:33450. doi: 10.1038/srep33450 (PMC5028837; doi:10.1038/srep33450)
Supplement: Supplementary Information [file srep33450-s1.doc]

**Supplementary data**

**Synthesis and Evaluation of ^18^F-labeled Pyridaben Analogues for Myocardial Perfusion Imaging in Mice, Rats and Chinese mini-swine**

Tiantian Mou^1, 2#^, Zuoquan Zhao^3#^, Linyi You^1^, Yesen Li^1^, Qian Wang^2^, Wei Fang^3*^, Jie Lu^4^, Cheng Peng^5^, Xianzhong Zhang^1*^

1. Center for Molecular Imaging and Translational Medicine, State Key Laboratory of Molecular Vaccinology and Molecular Diagnostics, School of Public Health, Xiamen University, Xiamen 361005, China;

2. Department of Nuclear Medicine, Beijing Anzhen Hospital, Capital Medical University, Beijing 100029, China;

3. Department of Nuclear Medicine, Car­diovascular Institute and Fu Wai Hospital, Chinese Academy of Medical Sciences, Beijing 100037, China;

4. Key Laboratory of Radiopharmaceuticals, Ministry of Education, College of Chemistry, Beijing Normal University, Beijing 100875, China;

5. PET Center, Xuanwu Hospital of Capital University of Medical Sciences, Beijing, 100053, China.

**1.** **The characterization data of the tosylated precursors**

^1^H NMR for mpp1-OTs: ^1^H NMR, CDCl_3_ (400 MHz, CDCl_3_) δ: 1.637 (s, 9H, N(C*H_3_*)*_3_*), 2.435 (s, 3H, phenyl-C*H_3_*), 3.722 (d, 2H, C*H_2_*CH_2_OS), 4.150 (d, 2H, CH_2_C*H_2_*OS), 4.651 (s, 2H, triazolyl-C*H_2_*-O), 5.288 (s, 2H, phenyl-C*H_2_*-O), 5.571 (s, 2H, phenyl-C*H_2_*- triazolyl), 7.306-7.456 (m, 6H, *phenyl*), 7.573 (s, 1H, N=C-*H*), 7.717 (s, 1H, *triazolyl*), 7.750 (d, 2H, *phenyl*)。

^1^H NMR, ^13^C NMR, IR and ESI-MS for mpp2-OTs: ^1^H NMR (400 MHz, CDCl_3_) δ: 1.637 (s, 9H, N(C*H_3_*)*_3_*), 2.436 (s, 3H, phenyl-C*H_3_*), 3.585-3.664 (m, 6H, OC*H_2_*C*H_2_*OC*H_2_*C*H*_2_OS), 4.130 (t, 2H, OCH_2_C*H_2_*OS), 4.692 (s, 2H, O-C*H_2_*-triazolyl), 5.271 (s, 2H, triazolyl-C*H_2_-*phenyl), 5.569 (s, 2H, O-C*H_2_*-C=C-Cl), 7.310 (d, 2H, CH_3_-*phenyl*), 7,331-7.7.768 (m, 8H, *phenyl*-CH_2_, *triazolyl*, N=C-*H*, CH_2_-*phenyl*); ^13^C NMR (100 MHz; CDCl_3_) δ: 21.60, 27.81, 53.71, 60.32, 64.67, 66.39, 68.57, 69.62, 70.57, 71.33, 118.10, 122.81, 124.97, 126.65, 127.30, 127.84, 128.35, 129.66, 132.79, 135.62, 135.91, 144.91, 145.56, 153.56, 158.91, 162.47; IR (CH_2_Cl_2_ cm^-1^) *v*: 1649 (C=O); ESI-MS calculated for C_30_H_36_ClN_5_O_7_S: 645.2, Found: 646.8.

^1^HNMR for mpp3-OTs: ^1^H NMR (400 MHz, CDCl_3_) δ: 1.640 (s, 9H, N(C*H_3_*)*_3_*), 2.439 (s, 3H, phenyl-C*H_3_*), 3.558-3.695 (m, 10H, (OC*H_2_*C*H_2_*)*_2_*OC*H_2_*CH_2_OS), 4.131 (t, 2H, OCH_2_C*H_2_*OS), 4.693 (s, 2H, O-C*H_2_*- triazolyl), 5.275 (s, 2H, triazolyl-C*H_2_-*phenyl), 5.559 (s, 2H, C*H_2_*-O-C=C-Cl), 7.317 (d, 2H, CH_3_-*phenyl*), 7.337-7.760 (m, 8H, *phenyl*-CH_2_, *triazolyl*, N=C-*H*, CH_2_-*phenyl*).

**2. The characterization data of the non-radioactive compounds**

^1^H NMR, ^19^F NMR, and IR for [^19^F]Fmpp1: ^1^H NMR (400 MHz, CDCl_3_) δ: 1.640 (s, 9H, N(C*H_3_*)*_3_*), 3.786 (dt, 2H, C*H_2_*CH_2_F); 4.562 (dt, 2H, CH_2_C*H_2_*F), 4.708 (s, 2H, triazolyl-C*H_2_*-O), 5.283 (s, 2H, phenyl-C*H_2_*-O), 5.561 (s, 2H, phenyl-C*H_2_*- triazolyl), 7.287-7.541 (m, 5H, *phenyl*, *triazolyl*), 7.718 (s, 1H, N=C-*H*); ^19^F NMR δ: -223.15; IR (CH_2_Cl_2_ cm^-1^) *v*: 1647 (C=O);

^1^H NMR, ^13^C NMR, ^19^F NMR, and ESI-MS for [^19^F]Fmpp2: ^1^H NMR (400 MHz, CDCl_3_) δ: 1.641 (s, 9H, N(C*H_3_*)*_3_*), 3.679 (dt, 6H, OC*H_2_*C*H_2_*OC*H_2_*CH_2_F); 4.535 (dt, 2H, CH_2_C*H_2_*F), 4.703 (s, 2H, triazolyl-C*H_2_*-O), 5.276 (s, 2H, phenyl-C*H_2_*-O), 5.549 (s, 2H, phenyl-C*H_2_*- triazolyl), 7.249-7.558 (m, 5H, *phenyl*, *triazolyl*), 7.709 (s, 1H, N=C-*H*); ^13^C NMR (100 MHz; CDCl_3_) δ: 27.75, 27.97, 53.77, 64.67, 69.77, 70.32 (d, J=15 Hz), 70.49, 70.63, 83.08 (d, J=134 Hz), 118.17, 122.76, 124.97, 126.57, 127.37, 128.337, 128.80, 129.73, 135.56, 135.96, 153.57, 158.96; ^19^F NMR δ: -222.80; IR (CH_2_Cl_2_ cm^-1^) *v*: 1648 (C=O); ESI-MS calculated for C_23_H_29_ClFN_5_O_4_: 493.19, Found: 494.20.

^1^H NMR, ^19^F NMR, and ESI-MS for [^19^F]Fmpp3: ^1^H NMR (400 MHz, CDCl_3_) δ: 1.561 (s, 9H, N(C*H_3_*)*_3_*), 3.550-3.690 (m, 10H, O(C*H_2_*C*H_2_*O)*_2_*C*H_2_*CH_2_F), 4.459 (dt, 2H, C*H_2_*F), 4.600 (s, 2H, O-C*H_2_*-triazolyl), 5.210 (s, 2H, phenyl-C*H_2_*-O), 5.472 (s, 2H, phenyl-C*H_2_*-triazolyl), 7.188-7.498 (m, 5H, *phenyl*, *triazolyl*), 7.666 (s, 1H, N=C-*H*); ^19^F NMR δ: -222.74; ESI-MS calculated for C_25_H_33_ClFN_5_O_5_: 537.22, Found: 538.7., O-*phenyl*), 7.917 (d, 2H, CO- *phenyl*); IR (CH_2_Cl_2_ cm^-1^) *v*: 1712 (C=O), 1254 (=C-O-C), 1059 (C-O-C).

**3. Relationship between UV area and amount of [^19^F]Fmpp1, [^19^F]Fmpp2 and [^19^F]Fmpp3 loaded**

Figure S1. Relationship between UV area and amount of [^19^F]Fmpp1, [^19^F]Fmpp2 and [^19^F]Fmpp3 loaded.

**4. The SUV** **of radiotracers in Chinese mini-swine**

Table S1. The SUV of [^18^F]Fmpp1, [^18^F]Fmpp2, and [^18^F]Fmpp3 in Chinese mini-swine

| Tissues | Radiotracers | Post-injection time (min) | | | |
| --- | --- | --- | --- | --- | --- |
|  |  | 5 | 30 | 60 | 120 |
| Heart | [^18^F]Fmpp1 | 6.82 | 5.7 | 5.03 | 2.19 |
|  | [^18^F]Fmpp2 | 7.12 | 6.97 | 6.5 | 5.75 |
|  | [^18^F]Fmpp3 | 4.51 | 5.46 | 5.17 | 4.53 |
| Liver | [^18^F]Fmpp1 | 6.72 | 2.96 | 2.71 | 1.62 |
|  | [^18^F]Fmpp2 | 4.15 | 1.69 | 1.2 | 0.96 |
|  | [^18^F]Fmpp3 | 4.10 | 2.13 | 1.30 | 1.12 |
| Heart/Liver | [^18^F]Fmpp1 | 1.01 | 1.93 | 1.86 | 1.35 |
|  | [^18^F]Fmpp2 | 1.72 | 4.12 | 5.42 | 5.99 |
|  | [^18^F]Fmpp3 | 1.10 | 2.56 | 3.98 | 4.04 |

**5. The SUV** **of [^18^F]Fmpp2 in mice and rats**

Table S2. The SUV of [^18^F]Fmpp2 in mice and rats

| organs | Post-injection time (min) | | | | | |
| --- | --- | --- | --- | --- | --- | --- |
|  | 5 | | 30 | | 120 | |
|  | **mice** | **rats** | **mice** | **rats** | **mice** | **rats** |
| heart | 1.967 | 6.721 | 2.010 | 7.942 | 0.503 | 3.800 |
| blood | 0.897 | 1.704 | 0.777 | 2.837 | 0.362 | 1.700 |
| liver | 1.847 | 4.647 | 1.318 | 3.039 | 0.355 | 1.400 |
| lung | 0.265 | 0.460 | 0.230 | 0.267 | 0.135 | 0.304 |
| muscle | 0.117 | 0.193 | 0.175 | 0.253 | 0.191 | 0.784 |
| heart/blood | 2.19 | 3.94 | 2.59 | 2.80 | 1.39 | 2.24 |
| heart/liver | 1.07 | 1.45 | 1.52 | 2.61 | 1.42 | 2.71 |
| heart/lung | 7.42 | 14.6 | 8.74 | 29.77 | 3.73 | 12.5 |
| heart/muscle | 16.79 | 34.79 | 11.51 | 31.38 | 2.64 | 4.85 |

**6.** **MicroPET/CT images in wildtype mice and rats.**


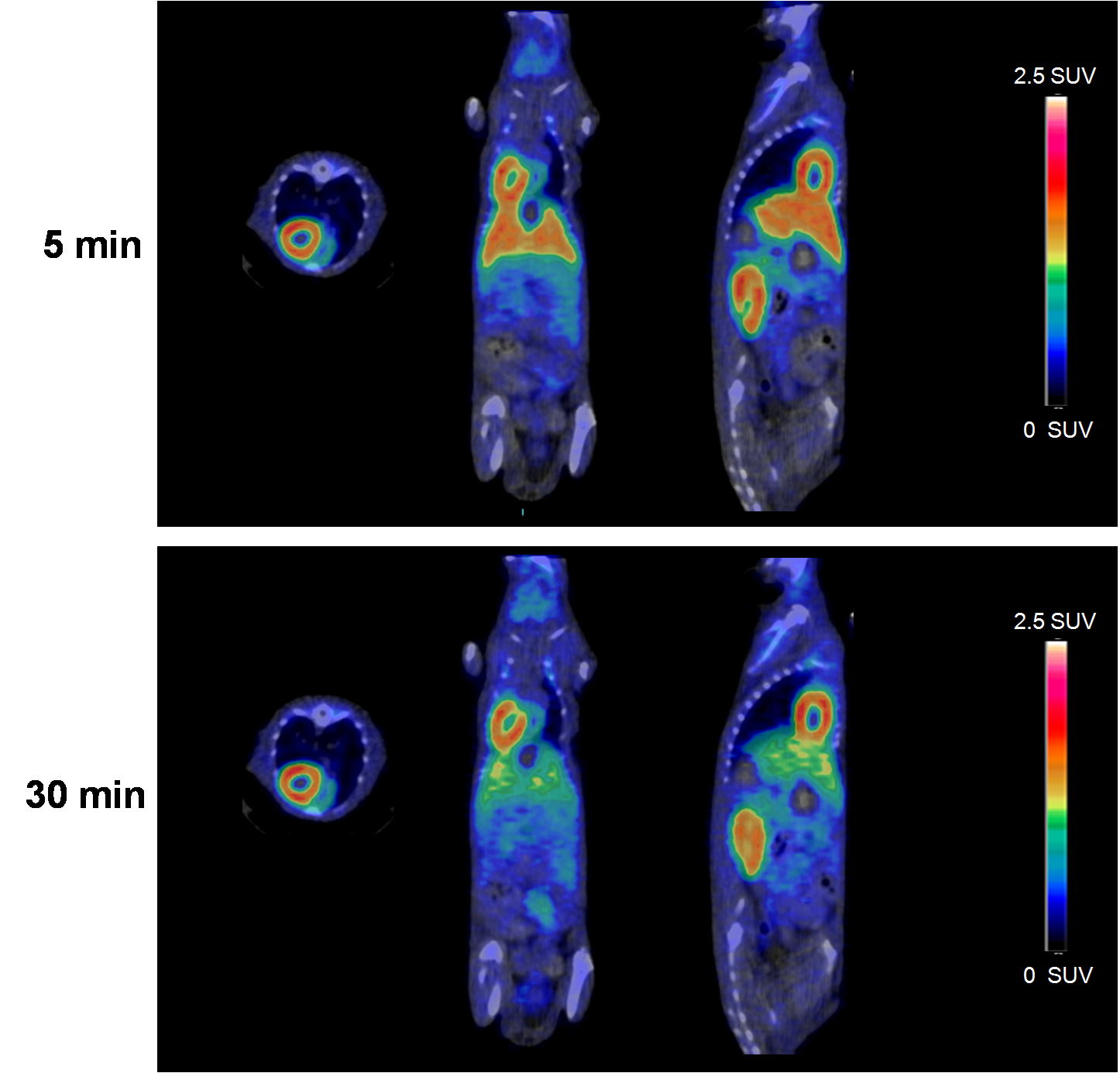


Figure S2. MicroPET/CT images in wildtype mice. Images were obtained with 10 MBq of [^18^F]Fmpp2 in 5% ethanol solution at 5 and 30 min after injection.


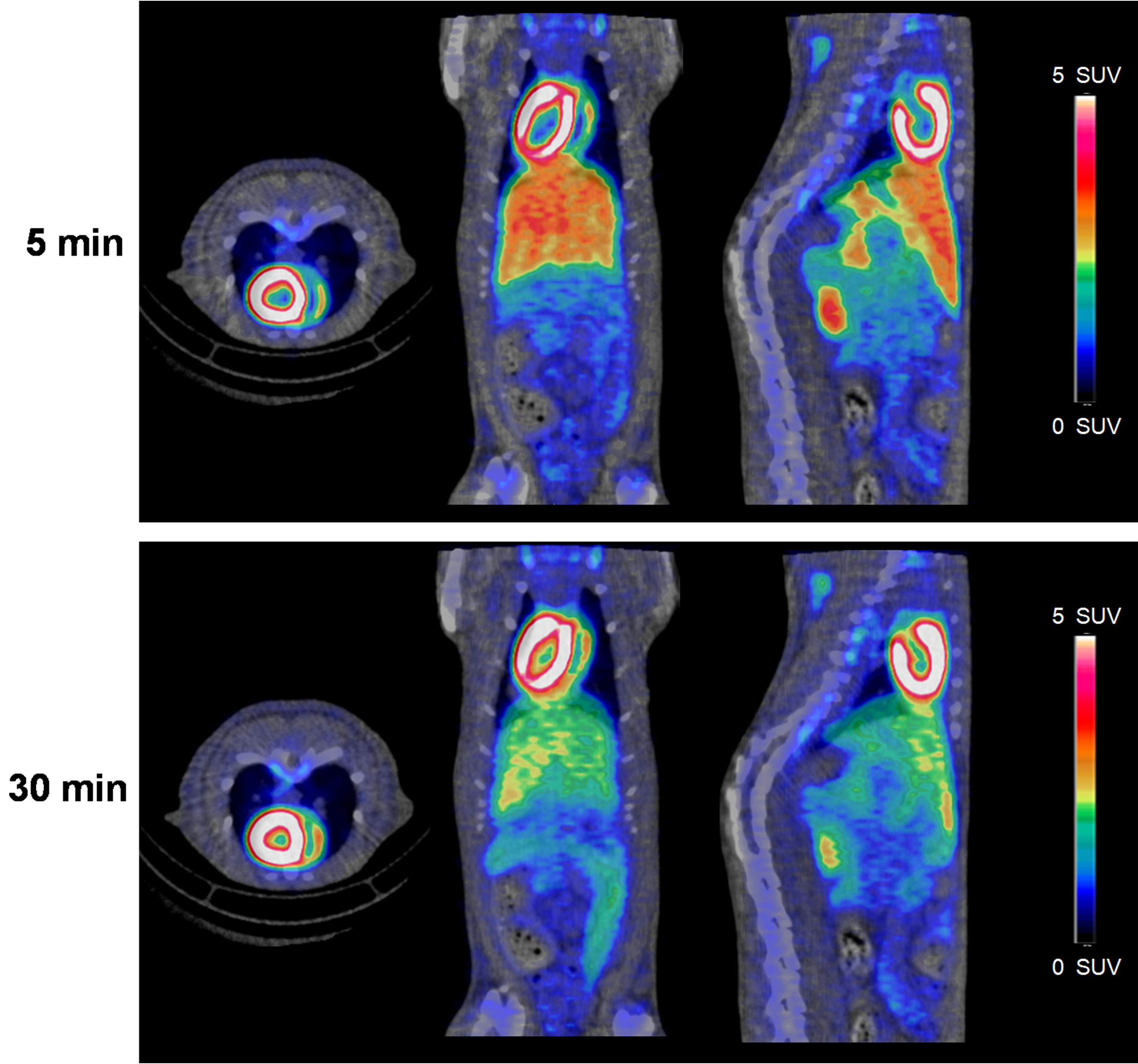


Figure S3. MicroPET/CT images in wildtype rats. Images were obtained with 35 MBq of [^18^F]Fmpp2 in 5% ethanol solution at 5 and 30 min after injection.

**7. Tissue** **uptake Time–Activity Curves (TACs) in wildtype mice.**


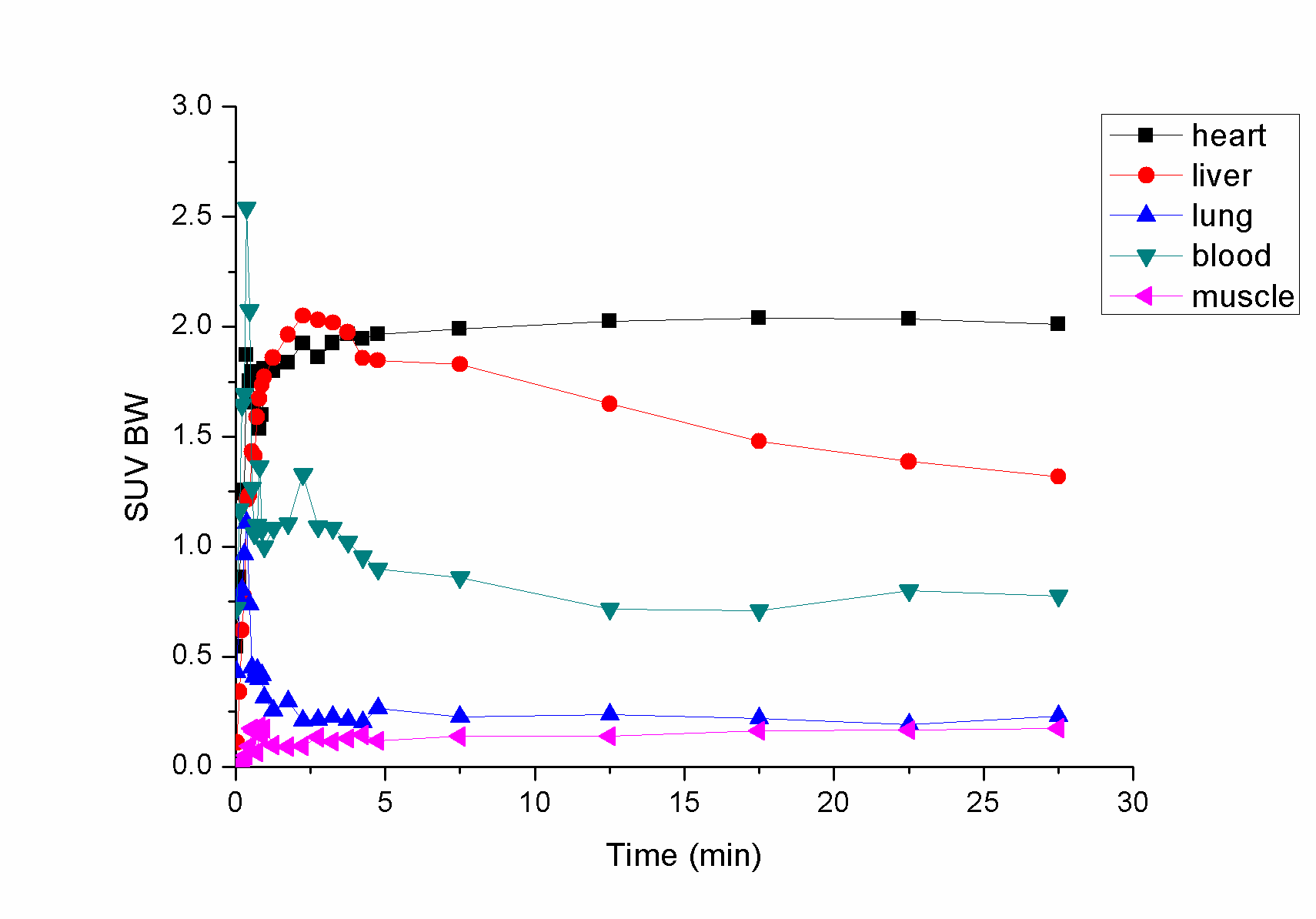


Figure S4. TACs derived from dynamic PET imaging of mice with 10 MBq of [^18^F]Fmpp2 in 5% ethanol solution.

**8. Tissue** **uptake Time–Activity Curves (TACs) in wildtype rats.**


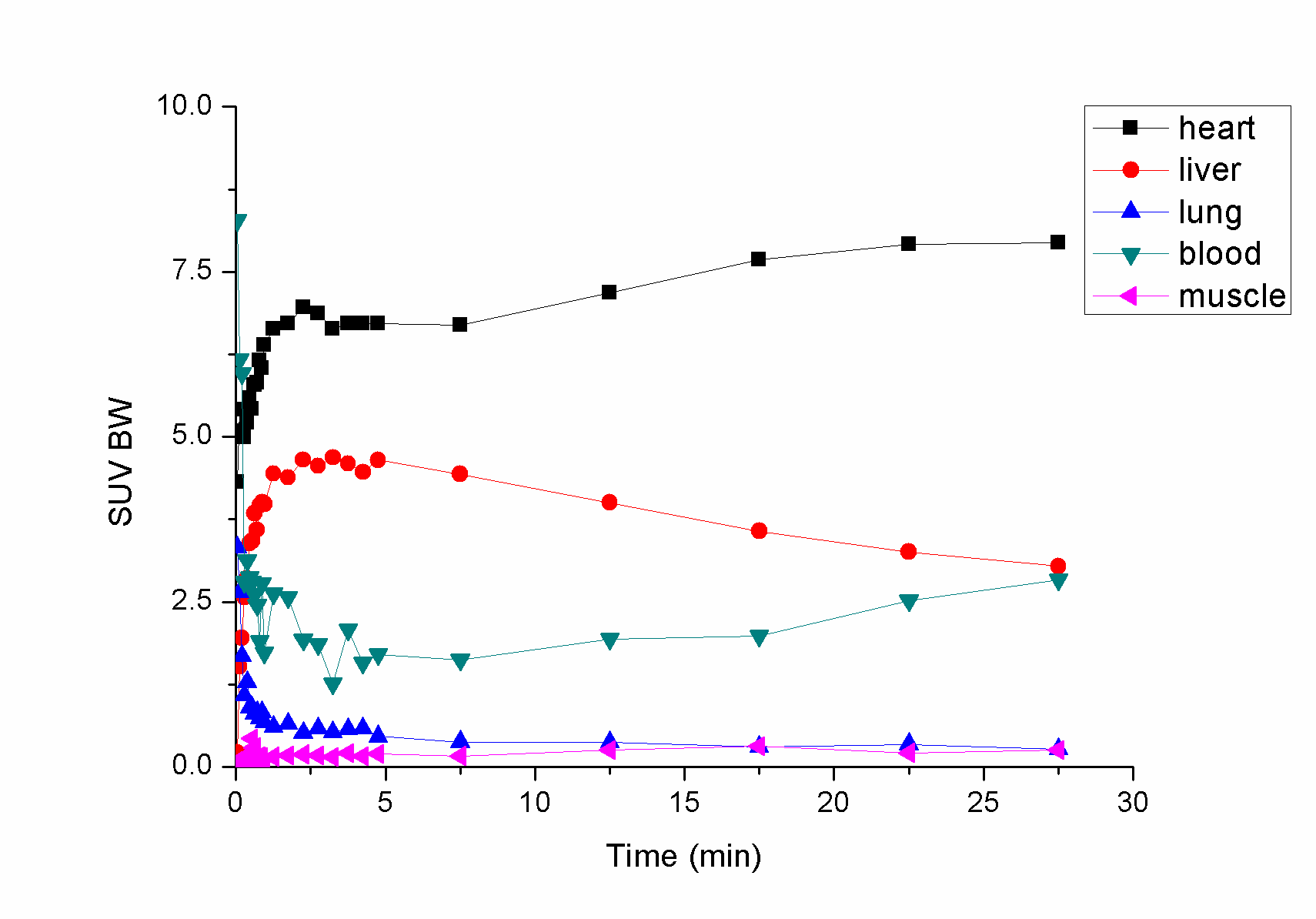


Figure S5. TACs derived from dynamic PET imaging of rats with 35 MBq of [^18^F]Fmpp2 in 5% ethanol solution.
